# Palmatine Ameliorates High-Temperature and High-Humidity-Induced Enteritis *via* Mitophagy and NLRP3 Inflammasome Degradation

**DOI:** 10.2174/0118715303348448250605071743

**Published:** 2025-07-15

**Authors:** Xiaoying Mo, Tai Mi, Wanli Liu, YaoWang, Yalan Wu, Xinhua Huang, Song Chen, Huanhuan Luo

**Affiliations:** 1 College of Traditional Chinese Medicine, Changsha Medical University, Changsha 410219, Hunan Province, China;; 2 Changsha Medical University, College of Traditional Chinese Medicine, Changsha, China; Present Address: Taoyuan Hospital of Traditional Chinese Medicine, Changde 415700, Hunan Province, China;; 3 School of Basic Medicine, Guangzhou University of Chinese Medicine, Guangzhou 510006, Guangdong Province, China;; 4 Science and Technology Innovation Center of Guangzhou University of Chinese Medicine, Guangzhou 510006, Guangdong Province, China;; 5 State Key Laboratory of Dampness Syndrome of Chinese Medicine, Guangzhou, Guangdong 510006, China

**Keywords:** NLRP3, interleukin-1β (IL-1β), mitochondrial DNA (mtDNA) copies, inflammatory bowel disease, HTH-induced enteritis, targeted genes

## Abstract

**Background:**

Previous studies have shown that a High-Temperature- and High-Humidity (HTH) environment leads to mild enteritis, accompanied by impaired mitophagy and activated NLRP3-IL-1β, as found in Inflammatory Bowel Disease (IBD). Therefore, whether Palmatine (PAL), a candidate for treating IBD, ameliorates HTH-induced enteritis *via* mitophagy-associated mechanisms was examined. This study aimed to investigate the protective effects of PAL against HTH-induced enteritis in mice and determine the underlying mechanisms.

**Methods:**

BALB/c mice were used to model HTH-induced enteritis. The mice were exposed to an HTH environment (33 ± 0.5°C, 85-90% humidity) for 28 days, with PAL or Cyclosporin A (CsA) administered daily. Mice were euthanized on days 7, 14, 28, or 35 to analyze the ileal tissues. Pathological examination, western blotting (Parkin, NLRP3, IL-1β), immunofluorescence (8-OHDG), and mtDNA quantification were performed to assess the therapeutic effects of the treatment.

**Results:**

A total of 884 and 2,668 potential targeted genes were identified for PAL and IBD, respectively, including 183 overlapped genes, which were mainly involved in oxidative stress, inflammation, and autophagy. HTH induced weight loss and loose faeces, along with increased NLRP3, IL-1β, and 8-OHDG expressions, and decreased Parkin and mtDNA expressions in the ileum. These effects were ameliorated and exacerbated by PAL and CsA treatment, respectively.

**Discussion:**

Palmatine ameliorates HTH-induced enteritis by regulating the mitophagy-NLRP3 inflammasome axis. It activates Parkin-mediated mitophagy, increasing mtDNA copy number, reducing mtROS and 8-OHDG, thereby inhibiting NLRP3 inflammasome activation and IL-1β secretion. CsA reversed these effects, confirming the mitophagy-dependent mechanism. The safe dosage supports its therapeutic potential against environment-related intestinal inflammation.

**Conclusion:**

PAL ameliorated HTH-induced enteritis probably *via* augmenting mitophagy and in hibiting NLRP3 expression. The findings highlight the critical role of mitophagy in the pathogenesis of HTH-induced enteritis and support the potential use of PAL in treatment.

## 1 INTRODUCTION

Inflammatory Bowel Disease (IBD) is a group of chronic, recurrent, nonspecific inflammatory diseases. However, the aetiology of IBD has not yet been fully elucidated. The intestinal mucosa undergoes persistent inflammation due to immune dysregulation, causing intestinal barrier dysfunction, which may contribute to the pathogenesis of enteritis [1]. Intestinal bacterial disorders and the disruption of the intestinal mucosal barrier can allow the penetration of bacteria and their components into the intestine, activating the mucosal immune response, inducing inflammation, and exacerbating intestinal damage [2]. It was previously found that High Temperature and Humidity (HTH) can seriously dysregulate the intestinal microecology, increasing the levels of related proinflammatory cytokines, such as Interleukin (IL)-6 and tumour Necrosis Factor (TNF-α), in the intestinal vicinity, exacerbating local inflammation and leading to the development of enteritis [3]. Heat stress also causes microecological dysregulation and intestinal injury [4] and may be involved in the pathogenesis of IBD. Important roles are played in this process by Nod-Like Receptor (NLR) family pyrin-domain-containing 3 (NLRP3) and its associated inflammatory factors, such as IL-1β and IL-18 [5, 6].

NLRP3 is a member of the cytoplasmic NLR family that senses the presence of pathogens through recognizing pathogen-associated molecular patterns as well as endogenous risk and stress through danger-associated molecular patterns [7, 8]. NLRP3 is widely present in the epithelial cells of intestinal tissues and is crucial in intestinal inflammatory immunity. NLRP3 integrates with apoptotic speck-like protein, which contains a caspase recruitment domain (ASC) and procaspase-1 to constitute the NLRP3 inflammasome. NLRP3 activation promotes caspase-1 activation, cleaving the precursors of IL-1β and IL-18 into their mature forms, which are then secreted from cells to enhance the inflammatory response [9]. This process can lead to intestinal barrier dysfunction, which is associated with enteritis. NLRP3 inflammasomes are negatively regulated through mitophagy, which is a selective form of autophagy that degrades damaged or dysfunctional mitochondria and is essential for normal intracellular homeostasis as well as mitochondrial health. Mitophagy may regulate the inflammatory response of immune cells [10]. Therefore, mitophagy could be promoted to inactivate NLRP3 inflammasomes and prevent excessive inflammation. This strategy provides a new perspective for treating HTH-induced enteritis.

The treatments currently available for IBD mainly include anti-inflammatory and immunosuppressive drugs, such as aminosalicylic acid, glucocorticoid steroids, and thiopurines, which often have serious side effects. Antibody-based therapies, such as infliximab, and probiotics are effective. However, these drugs are expensive, which limits their widespread use [11]. *Coptis chinensis* is a goldthread flowering plant that is used as a common herbal medicine in China to relieve diarrhoea and gastrointestinal diseases. Clinical trials confirmed that traditional Chinese medicine prescriptions containing *C. chinensis*, such as the Coptis chinensis decoction, can improve clinical symptoms as well as biochemical test and pathological biopsy results in patients with enteritis [12]. The bioactive component isolated from this decoction, palmatine, is an isoquinoline alkaloid documented in the Chinese Pharmacopoeia as an anti-inflammatory drug [13]. Palmatine is used to alleviate dextran sodium sulphate-induced acute murine enteritis and inactivate NLRP3 inflammasomes in macrophages through enhancing PTEN-induced kinase 1/Parkin-driven mitophagy [14]. Palmatine can be clinically used for treating pelvic inflammation, abdominal pain, enteritis, and other conditions [15, 16]. Palmatine is a bioactive compound with anti-inflammatory, antioxidant, and antimicrobial properties. It is a candidate for therapeutic research owing to these properties, given the similarity in the pathogenesis between HTH-induced enteritis and Inflammatory Bowel Disease (IBD), such as inflammation, oxidative stress, and impaired autophagy. However, whether palmatine protects against High-Temperature and High-Humidity (HTH)-induced enteritis remains unclear. Therefore, the aim was to evaluate how HTH induces enteritis and the effects of palmatine on HTH-induced enteritis in mice, and elucidate the underlying mechanisms of these effects.

Network pharmacology differs from traditional single-target, single-drug pharmacological strategies by considering biological systems, drugs, and diseases from a network perspective, using a holistic philosophy similar to that of traditional Chinese medicine [17]. Therefore, network pharmacology was combined with experiments to identify the mechanisms through which palmatine acts in the treatment of IBD.

## 2 METHODS

### 2.1 Network Pharmacology Studies

#### 2.1.1 Common Target Screening for Palmatine and IBD

SuperPred is a prediction web server that provides anatomical therapeutic chemical codes and can be used for predicting the targets of compounds. SuperPred enables the prediction of the medical indication of novel compounds and the identification of new leads for known targets [18]. PharmMapper (http://www.lilab-ecust.cn/Pharmmapper/) was designed to identify target candidates for small-molecule probes (drugs, natural products, or other newly discovered compounds with unidentified binding targets) using a pharmacophore mapping approach [19]. GeneCards (https://www.genecards.org/Guide/CitaTions) is a human gene database, providing concise genomic, proteomic, transcriptional, genetic, and functional data for all known and predicted human genes [20]. Online Mendelian Inheritance in Man (OMIM) is a repository of comprehensive and selected information on genes, phenotypes, and their relationships. Diseases are linked to genes of the human genome in OMIM, which provides relevant references for further research and genome analysis tools for catalogued genes [21, 22]. DisGeNET is one of the largest available collections of genes and variants involved in human diseases, which is homogeneously annotated using controlled vocabularies and community-driven ontologies [23]. ‘Palmatine’ was used as the search term in the SuperPred and PharmMapper databases to obtain the corresponding targets. ‘Inflammatory bowel disease’ was used to retrieve 2,121 targets from GeneCards with correlation coefficients ≥ 10, and 547 and 265 targets were obtained from the OMIM and DisGeNET databases, respectively. We adopted the same correlation coefficient threshold of ±10 as adopted by Hu *et al*. in their network pharmacology analysis to screen potential targets [24], ensure consistency with their study, and retain the key targets related to the potential effects of palmatine.

The retrieved targets of palmatine and IBD were then uploaded to a Venn diagram production platform (https://bioinfogp.cnb.csic.es/tools/venny/index.html) to obtain the intersecting targets for the two terms. These were identified as the potential targets of palmatine in treating IBD.

#### 2.1.2 Gene Ontology (GO) and Kyoto Encyclopedia of Genes and Genomes (KEGG) Enrichment Analyses

The KEGG is a database resource for understanding advanced genomic, chemical, and system functions in biological systems (such as cells, organisms, and ecosystems). KEGG is used to systematically analyse the functions of genes to determine the genetic and chemical blueprints of biological phenomena. It is a manually created knowledge base that captures and organises experimental findings in a computable form [25]. The Database for Annotation, Visualisation, and Integrated Discovery (DAVID; https://david.Ncifcrf.gov/home.jsp) is a tool for analysing gene function. DAVID helps users apply bioinformatics analysis methods for batch gene and protein analyses to determine the function of genes or proteins. The potential targets of palmatine in treating IBD were identified using DAVID. *P*<0.05 was set at the threshold for identifying significantly enriched key GO and KEGG pathways.

#### 2.1.3 Assay and Visualisation of Network Relationships

Cytoscape_v3.9.1 was used to visualise the relationships between the targets of palmatine in treating IBD and selected GO-Biological Processes (BPs) and KEGG pathways.

### 2.2 Animal Model Development

BALB/C mice (6 weeks old, weighing 20-30 g) were obtained from China Zhuhai Baicheng Tong Biotechnology Co., Ltd. (Zhuhai, China; Certificate no. 44822700003283). The BALB/c mice were chosen owing to their well-documented use in immunological and inflammatory research. They provide an appropriate model for studying IBD and related conditions. Male mice were used to minimise the potential confounding effects of the oestrous cycle. All experimental mice were bred and maintained under specific pathogen-free conditions (Pharmacy Department, Zhongshan City Hospital of Traditional Chinese Medicine, Zhongshan, China). The mice were not genetically modified and had the standard BALB/c genotype. No previous procedures or treatments were performed on these mice before this study.

### 2.3 Ethical Approval

Animals were housed in sterile cages in a room maintained at 24 ± 2°C with an average humidity of 60 ± 1% and a 12/12 hours light: dark cycle. The mice had access to acidified water and rodent chow *ad libitum*. Acidified water is commonly used in animal experiments to control bacterial growth in water and prevent infection [26]. The mice were allowed 1 week to acclimate, and then 96 mice were randomly assigned to one of four groups (six per group). The mice in the Normal Control (NC) group were maintained as described above, whereas the mice in the HTH, HTH + palmatine (HTH + PAL), and HTH + CsA groups were transferred into a sterilised climate chambers (model: RXZ-158A, Ningbo Jiangnan Instrument Factory, Ningbo, China) located in the same room. The mice in each group were intragastrically administered treatment once per day. The mice in the HTH+PAL group were administered 0.2 mL of palmatine at 9 a.m. every day, and the mice in the HTH+CSA group were administered 0.2 mL of Cyclosporine A (CsA). The climate chamber conditions were set as follows: 33 ± 0.5 °C and 85-90% humidity. The mice were alternately subjected to HTH conditions during day and night to induce enteritis. The food and drinking water were sterilised and renewed each day to avoid bacterial contamination, given the rapid growth of bacteria under the high temperature and humidity conditions. The body weight and faeces consistency were recorded every other week. The mice were continuously housed in the HTH box for 4 weeks to evaluate the effect of the HTH environment on enteritis. The mice in each group were placed in a normal temperature and humidity environment from days 28-35, drug administration was stopped, and the mice were housed for 1 week under normal dietary conditions. The mice were euthanized on days 7, 14, 28, and 35. The animals were closely monitored for signs of pain or distress throughout the experiment. Appropriate analgesics were administered if any signs were observed as per the institutional animal care guidelines.

The sample size for each experimental group was determined based on practical considerations and previous similar studies. The sample size was not formally calculated *a priori*; the decision to use six mice per group was based on a balance among statistical power, ethical considerations, and resource constraints. Studies with similar experimental paradigms have typically used sample sizes ranging from three to eight mice per group. Six mice per group were selected, considering the precedent and the need to ensure adequate statistical power to detect meaningful differences between the groups. The age and sex requirements specified for the experimental model were met. The mice exhibited normal baseline health status upon arrival. They showed no signs of pre-existing gastrointestinal conditions or other systemic illnesses. All included mice underwent acclimatization and were randomly assigned to the study groups. The data were obtained from animals/experimental units that met the predefined inclusion criteria and showed no evidence of data entry errors, technical artifacts, or outliers beyond acceptable thresholds. The weights of the mice were ranked from lightest to heaviest, and then the random function in Excel was used (=RANDBETWEEN(0,150)) to randomly divide the mice into groups of six, for a total of 16 groups; 7NC, 14NC, 28NC, 35NC, 7HTH, 14HTH, 28HTH, 35HTH, 7HTH+PAL, 14HTH+PAL, 28HTH+PAL, 35HTH+ CSA, 14HTH+CSA, 28HTH+CSA, and 35HTH+CSA.

### 2.4 Drugs, Reagents, and Antibodies

Palmatine was provided by Dawff Biotech, Ltd. (catalogue number: DASF1221Z; Nanjing, China). CsA was manufactured by Yantong Biological Products Co., Ltd. (catalogue number: B1922; Guangzhou, China). Forty mg of palmatine and CsA powder was weighed and dissolved in distilled water to a volume of 10 mL to prepare 4 mg/mL solutions according to the dosage volume (0.1 mL/10 g) once per day [27]. Mouse anti-IL-1β (12703), anti-Parkin (2132), and anti-tubulin (2148) antibodies were purchased from Cell Signaling Technology (Danvers, MA, USA). Mouse anti-NLRP3 antibodies (768319) were obtained from R&D Systems (Minneapolis, MN, USA). Mouse anti-8-hydroxydeoxyguanosine antibodies (8-OHDG; 62623) were purchased from Abcam (Cambridge, UK). A tissue genomic DNA extraction kit was purchased from Tiangen Biochemical Technology Co., Ltd. (Beijing, China).

### 2.5 Faecal Water Content and Mobility Assessment

Faecal water content is an indicator of gastrointestinal function and intestinal inflammation. Locomotor activity was recorded and quantified using small animal autonomic actigraphy to assess the mobility and overall activity level of the mice. The mice in each group were placed in a YLS-1C small animal autonomic actigraphy, and activity was recorded for 5 minutes. The fresh faeces of the mice in each group were collected within 2 hours and wet-weighed. The faeces were then dried at 70 °C for 24 hours and weighed again. The water content of the faeces was calculated as (faecal wet weight - faecal dry weight) / faecal wet weight.

### 2.6 Haematoxylin and Eosin Staining of Ileal Tissue

The tissue morphology, including the epithelial cell integrity, arrangement of the intestinal villi, and inflammatory cell infiltration, were examined using haematoxylin and eosin staining. The partial ileum was fixed in 10% formalin, embedded in paraffin, and cut into 4 μm-thick sections. The sections were stained with haematoxylin and eosin, and the histopathology was observed under a microscope.

### 2.7 Western Blotting Analysis

Protein levels were measured using western blotting to evaluate the levels of NLRP3 inflammasome and interleukin-1β. Parkin protein levels were measured to evaluate mitophagy activity and mitochondrial quality. The western blotting assays were performed using 10 mg of a frozen ileum sample. The samples were homogenised in 1 mmol/L phenylmethylsulfonyl fluoride (Biyuntian, China), 1× protease inhibitor cocktail (MedChemExpress, Monmouth Junction, NJ, USA; HY-K0010), 1× phosphatase inhibitor (MedChemExpress, HY-K0021), and radioimmunoprecipitation assay lysis buffer (Biyuntian). Proteins were quantified using the bicinchoninic acid assay method. Proteins were separated using SDS-PAGE at 50 μg/lane and transferred after 2 hours at 200 mA to polyvinylidene fluoride membranes (Millipore Corp., Burlington, MA, USA). The membranes were blocked with 5% non-fat powdered milk (Biorad, Hercules, CA, USA) for 2 hours at room temperature and then incubated with anti-IL-1β, -Parkin, and -NLRP3 primary antibodies overnight at 4 °C. The membranes were washed and then incubated with horseradish-peroxidase-conjugated secondary antibodies (Thermo Fisher Scientific Inc., Waltham, MA, USA) for 2 hours at room temperature. The samples were stained using an enhanced chemiluminescence assay kit (Thermo Fisher Scientific Inc.) and quantified with the ImageJ software (National Institutes of Health, Bethesda, MD, USA).

### 2.8 Immunofluorescence Assay

Specific cellular markers, such as 8-OHDG, were detected and quantified to evaluate oxidative stress and mitochondrial damage. Part of the ileum was fixed with 4% formalin, embedded in paraffin, and cut into 4 μm-thick sections. The slides were placed in an incubator, baked at 60 °C for 1 hour, dewaxed, hydrated, sealed with Triton-X-100 (Beyotime Biotechnology, Nantong, China) for 10 minutes, and washed with phosphate-buffered saline with Tween 20 (PBST). Antigens were retrieved with a citrate/ethylenediaminetetraacetic acid solution. The sections were sealed with 3% H_2_O_2_ for 15 minutes, washed with PBST, sealed with immunofluorescent blocking solution for 30 minutes, and incubated with anti-8-OHDG primary antibodies at 4°C overnight. The sections were washed with PBST and incubated with Alexa-Fluor-555- or 488-conjugated IgG antibodies at 37°C for 1 hour. The sections were then incubated with 4′,6-diamidino-2-phenylindole (DAPI; 10 μg/mL) for 10 minutes. Images were acquired using a laser-scanning confocal microscope. Four slices were randomly selected, and the fluorescence intensity was calculated with the ImageJ software.

### 2.9 Mitochondrial DNA Testing

The mitochondrial DNA copy number was quantified to assess mitochondrial function and integrity. An appropriate amount of frozen intestinal tissue was cut with a sterile blade, and DNA was extracted using a tissue genome DNA extraction kit (Tiangen Biochemical Technology Co., Ltd.). The following primers were used: COXIII, 5′-CAATTACATGAGCTCATCATAGC [F] and 5′-CCATGGAATCC AGTAGCCA [R]; B2M, 5′-ATCCAAATGCTGAAGAACGG [F] and 5′-ATCAGTCTCAGTGGGGGTGA [R]. A real-time PCR (Polymerase Chain Reaction) assay (TB Green^TM^ Premix ExTaq^TM^ II; Takara Biotechnology, Dalian, China) was used to perform two independent PCR reactions for each DNA sample. The reactions were detected using an ABI 7500 Real-Time PCR System (Applied Biosystems, Foster City, CA, USA). The mitochondrial DNA (mtDNA) copy number was calculated using the relative quantification method, mtDNA relative copy number = 2-[CTCoxIII - CTB2M]. Ultimately, the copy number ratio between each group and the NC group was calculated.

### 2.10 Statistical Analysis

The SPSS 20.0 software was used for analysing the data, and the experimental data were subjected to the Shapiro-Wilk normality test and Levene’s homogeneity test of variance. Normally distributed continuous data are expressed as mean ± standard deviation. Data that met the homogeneity requirement of variance were compared using one-way ANOVA, and the Least Significant Difference (LSD) test was used for multiple comparisons between groups. Data that did not meet the homogeneity requirement of variance were subjected to the Tamhane T2 test for multiple comparisons between groups. The Kruskal-Wallis test was used for multiple comparisons between groups that did not conform to the normal distribution, and a P < 0.05 was considered a significant difference.

## 3 RESULTS

### 3.1 Common Targets for IBD and Palmatine

The search term ‘palmatine’ was used to identify 365 and 557 targets common to palmatine and IBD from the SuperPred and PharmMapper databases, respectively, which totalled 884 targets after removing duplicates. In addition, 2,121 targets with correlation coefficients > 10.00 were obtained from GeneCards using ‘inflammatory bowel disease’ as the search term. A total of 547 and 265 known therapeutic targets for IBD were obtained from the OMIM and DisGeNET databases, respectively, for a total of 2,668 therapeutic targets for IBD. Venny 2.1.0 was used to construct a Venn diagram by localising the 2,668 therapeutic targets of IBD to the 884 common targets of palmatine, which is shown in Fig. (**1**). A total of 183 target genes were finally identified as common target genes for IBD and palmatine.

### 3.2 Common Targets Enriched in GO and KEGG BP Pathways

**Fig. (2):**
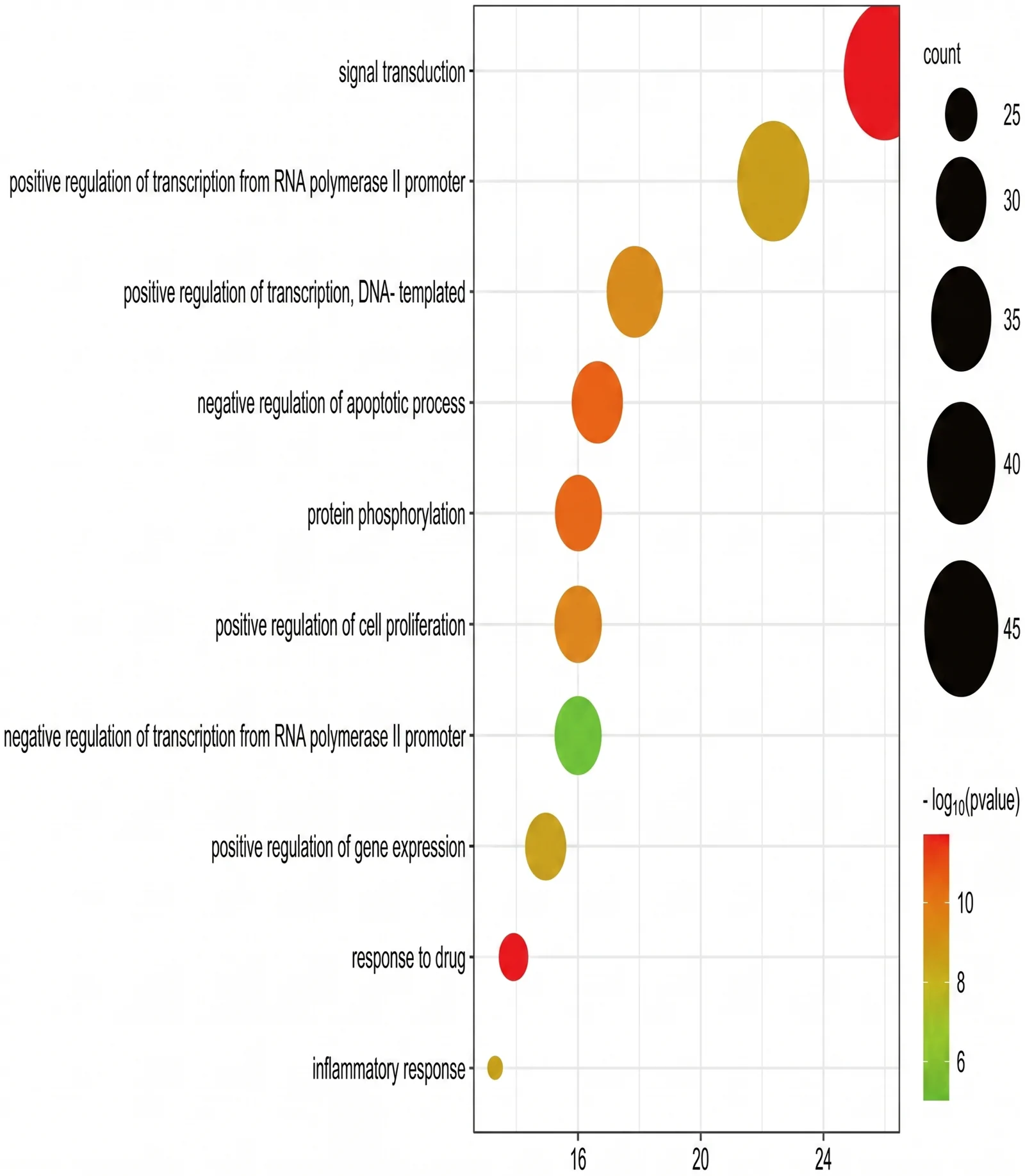
Top 10 biological processes through which IBD may be treated with palmatine.

GO and KEGG analyses of the 183 common targets were performed using the DAVID database to determine ontology clustering. The GO BP pathways associated with these targets mainly included (Fig. **2**) signalling, positive transcription of RNA polymerase II promoter, positive regulation of transcription, DNA template, negative regulation of the apoptotic process, protein phosphorylation, positive regulation of cell proliferation, negative regulation of RNA polymerase II promoter transcription, positive regulation of gene expression, drug response, and inflammatory response. KEGG pathway enrichment analysis was performed to further identify and analyse the mechanistic network of the complex regulatory genes. Enrichment was found in the following pathways: PI3K-AKT signalling, MAPK signalling, lipids and atherosclerosis, proteoglycans in cancer, chelate oncogenic-reactive oxygen species, Th17 cell differentiation, RAS signalling, RAP1 signalling, and hepatitis B, as shown in Fig. (**3**).

### 3.3 Palmatine Network for Treating IBD

**Fig. (4):**
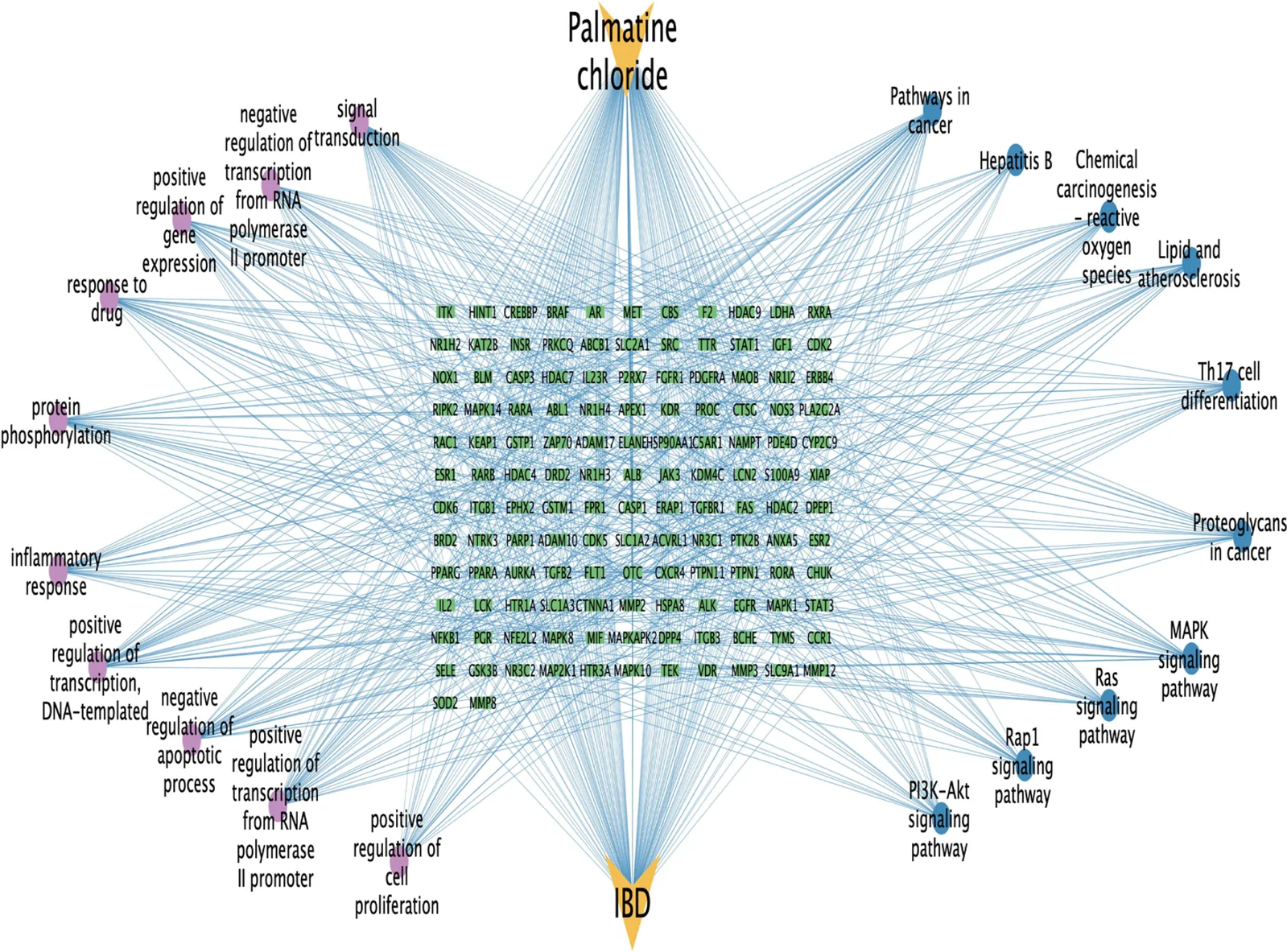
Detailed integrated visualisation of the network of the interactions of palmatine, its targets, Gene Ontology pathways, Kyoto Encyclopedia of Genes and Genomes pathways, and IBD.

The data from the top 10 enriched GO terms and KEGG pathways was used to create a network to visualise the palmatine-target-IBD interactions using Cytoscape_v3.9.1 (shown in Fig. **4**). Palmatine was found to share 10 BPs and 10 KEGG pathways with IBD, highlighting its potential as a target for treating IBD with palmatine.

### 3.4 Palmatine Alleviated Symptoms of HTH-Induced Enteritis in Mice

**Fig. (5):**
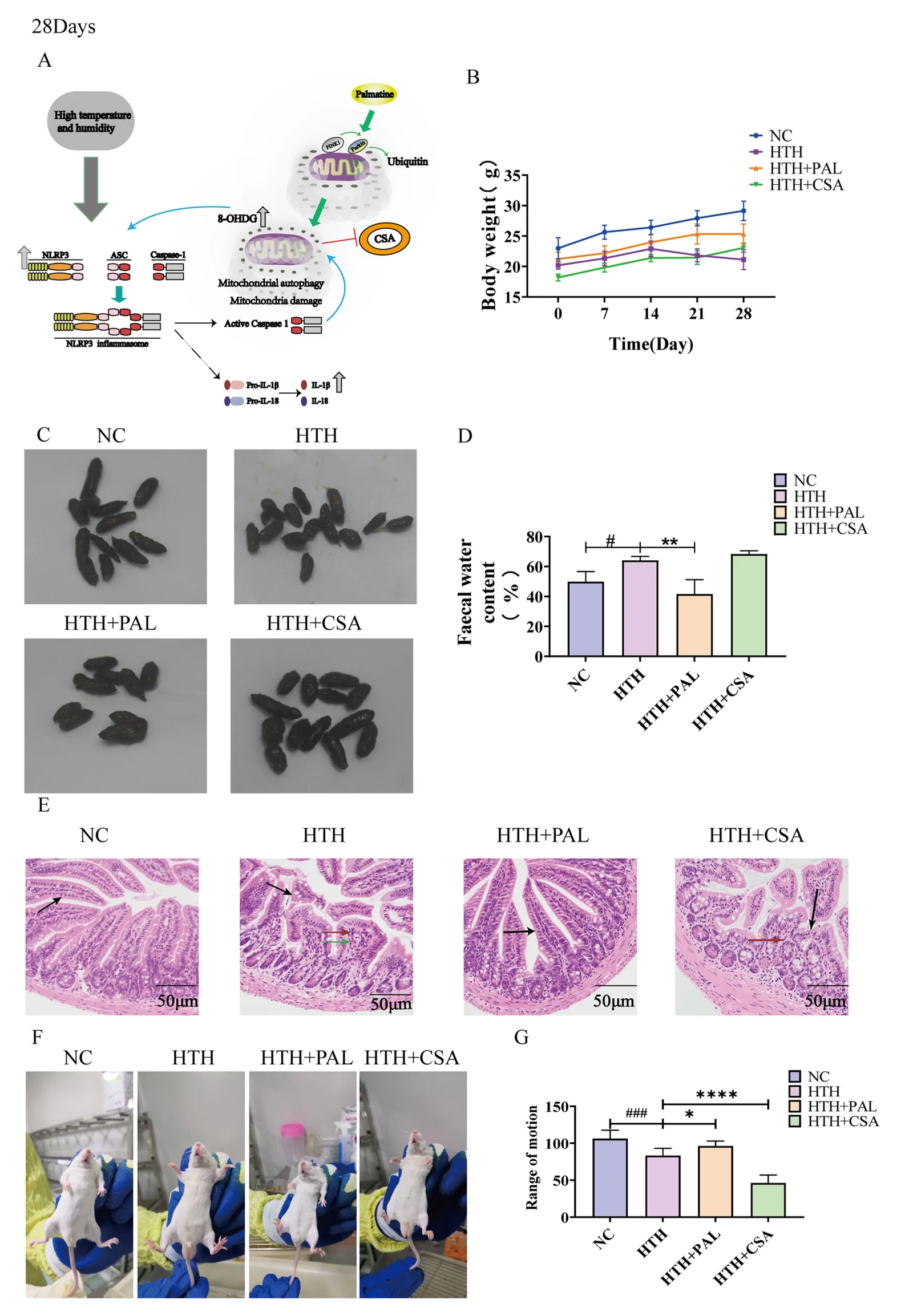
Schematic diagram of experimental design, mouse weight, faecal appearance, faecal water content, ileal HE histological changes, scrotal prolapse changes, and mobility. (**a**) Schematic diagram of the experiment. (**b**) Profile of weight (mean ± standard deviation, n = 6). (**c**) Appearance of faeces. (**d**) Changes in faecal water content (n = 6). (**e**) Representative haematoxylin and eosin (H&E) staining images of ileal tissue (scale bar, 50 μm, n=3). (**f**) Changes in scrotal sagging(n=3). (**g**)range of motion(n=6). #:*P* < 0.05 *vs*. NC group; ###:*P* < 0.001 *vs*. NC group; *: *P* < 0.05 *vs*. HTH group; **:*P* < 0.01 *vs*. HTH group; ****: *P* < 0.0001 *vs*. HTH group. Mean ± standard deviation, one-way ANOVA, Kruskal-Wallis test.

A diagram of the experimental design is shown in Fig. (**5a**). The mice in the HTH group exhibited a gradually increasing reluctance to move, an unkempt and dull coat (Figs **5f** and **5g**), and a drooping scrotum compared to the mice in the NC group during the experimental period. These symptoms improved in the HTH group after administering palmatine. The mice in the HTH group lost weight (Fig. **5b**), produced sticky faeces (Fig. **5c**), with increased faecal water content after 28 days (Fig. **5d**). These symptoms worsened after administering CsA. In contrast, the faecal water content decreased, and the body weight gradually recovered after administering palmatine. In addition, histopathological analysis of ileum disclosedthat the mice in HTH group had enteritis (Fig. **5e**), indicated by the destroyed epithelial cells in the mucosal layer, a disturbed arrangement of the intestinal villi (black arrows), inflammatory cell infiltration (red arrows), and a small amount of capillary congestion and oedema (green arrows). Administering CsA also aggravated the pathological changes associated with enteritis. In contrast, administering palmatine healed the damage to the intestine.

### 3.5 Palmatine Attenuates Intestinal Inflammation and Inhibits the Activation of NLRP3 Inflammasomes in Mice Under HTH Conditions

As shown in Figs. (**6a** and **6b**), compared with the NC group, the HTH group showed increased NLRP3 protein levels in the ileal tissue, which caused the release of IL-1β during the 28 days of developing the model. CsA aggravated this phenomenon, whereas palmatine seemed to reverse NLRP3 and IL-1β protein levels in the ileal tissue of mice in the HTH group to a certain extent and alleviated enteritis at all time points.

### 3.6 Palmatine Promoted Parkin-Driven Mitophagy in Mice Under HTH Conditions

**Fig. (7):**
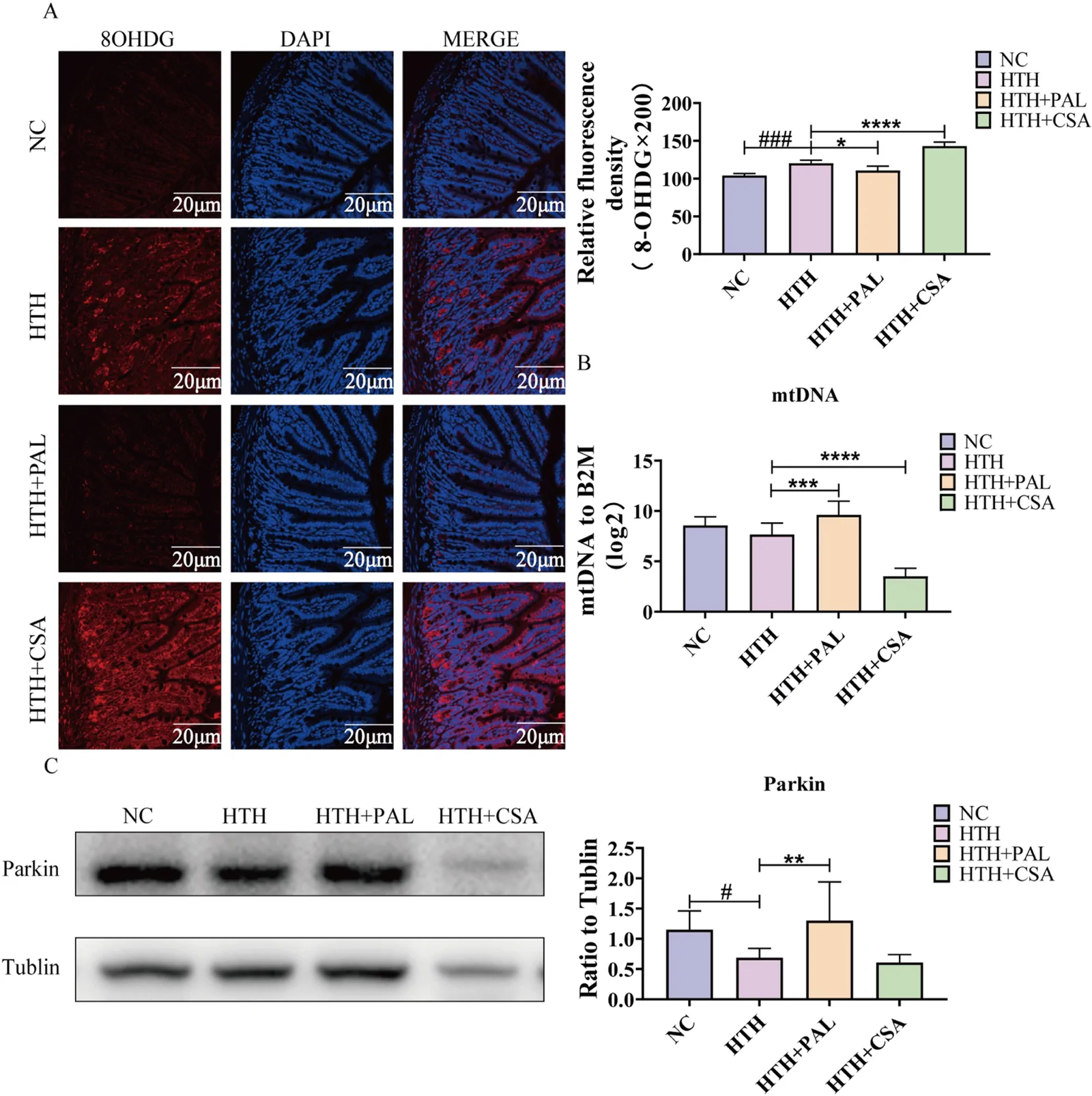
Palmatine promotes Parkin-mediated mitophagy and reduces mtDNA damage and 8-OHDG levels in mice under HTH conditions. (**A**) Ileal tissues were stained with 4′,6-diamidino-2-phenylindole (DAPI, blue) and immunostained for 8-hydroxydeoxyguanosine (8-OHDG; red) and observed under a confocal laser-scanning microscope (scale bar, 20 μm; n = 4). Quantification of 8-OHdG immunofluorescence (Fig. a, right, n=4). (**B**) Polymerase chain reaction detection of mitochondrial DNA (mtDNA) copy number in ileal tissues (n = 6). (**C**) Analysis of Parkin protein levels in mouse ileal tissues by western blotting (n = 6). #: *P* < 0.05 *vs*. the NC group; ###: *P* < 0.001 *vs*. the NC group; *: *P* < 0.05 *vs*. the HTH group; **: *P* < 0.01 *vs*. the HTH group; ***: *P* < 0.001 *vs*. the HTH group; ****: *P* < 0.0001 *vs*. the HTH group. Mean± standard deviation, one-way ANOVA, Kruskal-Wallis test.

The oxidative stress marker, 8-OHDG, was detected using immunofluorescence to determine whether palmatine inhibited NLRP3 inflammasome activation in the ileal tissue of mice in the HTH group through promoting mitophagy. The 8-OHDG fluorescence intensity increased in the HTH groups compared with those in the control after 28 days, suggesting the occurrence of oxidative stress (Fig. **7a**). The extent and intensity of 8-OHdG staining revealed oxidative damage and inflammation in the HTH mice (Fig. **7a**, right). Administering CsA induced high-intensity 8-OHDG fluorescence. However, administering palmatine decreased 8-OHDG fluorescence intensity and inhibited oxidative damage in the ileal tissue of the HTH-conditioned mice.

The mtDNA copy number was quantified using quantitative PCR with *B2M* as the internal reference gene, which allowed the comparison of mtDNA between the groups, to examine the effect of palmatine on mitochondrial function. The mtDNA copy number was lower in all HTH groups than in the NC group after 28 days (Fig. **7b**). CsA administration further reduced the mtDNA copy number. In contrast, administering palmatine increased the mtDNA copy number.

Western blotting was used to detect the levels of Parkin, a mitophagy-associated protein, in the ileal tissue samples. The protein level of Parkin was lower under HTH conditions than that in the NC group after twenty-eight days (Fig. **7c**). Administering CsA blocked mitophagy, leading to lower levels of Parkin in the CsA groups than in the HTH group. In contrast, administering palmatine increased Parkin levels and autophagic activity compared with those in the HTH group.

### 3.7 Persistence of Palmatine Efficacy

**Fig. (8):**
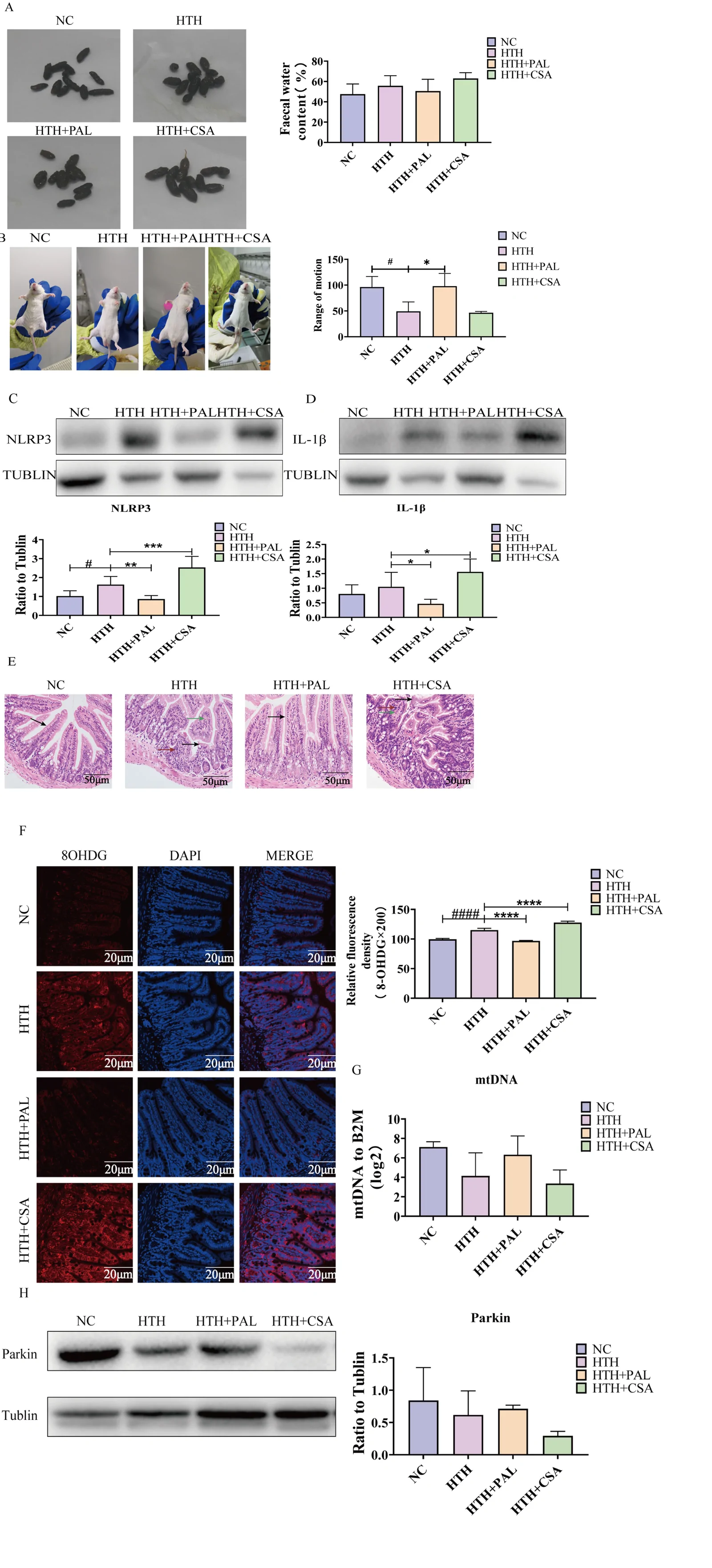
On day 7 of the experiment, following the cessation of treatment and removal from the HTH environment, changes in the faecal water content and appearance, histological changes in the ileum, NLRP3, IL-1β, and Parkin protein levels, changes in the mtDNA copy number, and 8-OHDG levels were evaluated. **a** Appearance of faeces and changes in the faecal water content (n = 6). (**b**) Scrotal droop (n=3) and changes in range of motion (n=6).)(**c**) NLRP3 protein levels in ileal tissue (n = 6). (**d**) IL-1β protein levels in ileal tissue (n = 6). (**e**) Representative H&E staining images of ileal tissue (scale bar, 50 μm, n=3). (**f**) Ileal tissues were stained with DAPI (blue), immunostained with 8-OHDG (red) and observed under a confocal laser-scanning microscope (scale bar, 20 μm; n = 4). Quantification analysis 8-OHdG immunofluorescence (panel right, n=4). (**g**) PCR analysis of mtDNA copy number in the ileal tissue (n = 6). (**h**) Analysis of Parkin protein levels in mouse ileal tissues by western blotting (n = 6). #: *P* < 0.05 *vs*. the NC group; ####: *P* < 0.0001 *vs*. the NC group;*: *P* < 0.05 *vs*. the HTH group; **: *P* < 0.01 *vs*. the HTH group; ***: *P* < 0.001 *vs*. the HTH group; ****: *P* < 0.0001 *vs*. the HTH group. Mean± standard deviation, one-way ANOVA, Kruskal-Wallis test.

The faecal water content was higher in the HTH group than in the NC group after removing the mice from the HTH environment and ceasing treatment for 7 days (day 35 of the experiment), although the difference in faecal water content was not significant (Fig. **8a**). Scrotal droop and mobility remained lower in the HTH group than in the NC group (Fig. **8b**). The pathological changes associated with enteritis in the HTH group, including the destruction of the epithelial cells in the mucosal layer, disorganised arrangement of the intestinal villi (black arrows), inflammatory cell infiltration (red arrows), and a small amount of capillary congestion and oedema (green arrows), did not improve after removing the mice from the HTH conditions (Fig. **8e**). The NLRP3 and IL-1β protein levels remained elevated (Figs. **8c** and **d**), and 8-OHDG fluorescence intensity remained higher in the HTH group than in the control (Fig. **8f**). The mtDNA copy number remained low (Fig. **8g**) and Parkin expression was suppressed (Fig. **8h**) in the HTH group compared with the control group. CsA administration also aggravated this phenomenon. In contrast, administering palmatine ameliorated the pathological damage caused by enteritis, reducing the NLRP3 and IL-1β protein levels, 8-OHDG fluorescence intensity, and oxidative damage in the ileal tissues of the mice in the HTH group. Administering palmatine also increased the mtDNA copy number, elevated the Parkin levels, and alleviated mitochondrial dysfunction, although the differences were not significant.

The indices in mice were also examined in the HTH + PAL group on days 7 and 14 of the experiment. Administering palmatine affected these indices on days 7 and 14, but the overall effect was not as strong as those observed on day 28. Therefore, the presented data are based on the results after the 28th day of palmatine treatment. The results for 7 and 14 days of treatment are shown in Additional Figs. (**1**-**4**).

## 4 DISCUSSION

IBD is a chronic inflammatory disease. The incidence and prevalence of IBD are the highest in developed countries, and the pathogenesis of IBD is related to genetic susceptibility and the immune response [28]. The results of our previous animal studies showed that HTH conditions lead to mild enteritis *via* increasing the expression of proinflammatory cytokines, including TLR4, NF-κB, IL-6, IL-1β, and ASC [29, 30]. Humidity, an improper diet, emotional disorders, and weakness due to chronic illness are thought to lead to dysregulation of gastrointestinal transportation, conduction disorders, blockage of qi, and venation obstruction, causing ‘dampness-heat enteritis’ in traditional Chinese medicine [31]. The pharmacology of biological targets and functional processes was determined to describe the pathways through which palmatine acts on IBD. Additionally, the mechanism through which HTH conditions induce enteritis through animal experiments was also evaluated. A new strategy was developed to treat environment-related discomfort through altering mitophagy. Palmatine shows potential as a therapeutic agent for treating IBD.

These findings are consistent with those of previous studies, indicating that the NLRP3 inflammasome plays a key role in intestinal inflammatory injury [32, 33]. The NLRP3 inflammasome acts as a potent inflammatory mediator that induces inflammation, inflammatory cell death, and the production of the proinflammatory cytokine IL-1β [34]. Moreover, these findings also showed that HTH conditions caused weight loss as well as increased faecal water content and inflammatory cell infiltration, resulting in pathological changes. HTH conditions increase the expression of NLRP3 and IL-1β, which trigger enteritis. Mitophagy reduces mtDNA accumulation and increases mitochondrial reactive oxygen species (mtROS) levels during NLRP3 inflammasome activation, negatively regulating NLRP3 inflammasome levels. This finding is consistent with those of Lin *et al*., who observed that similar changes in inflammation were induced by oxidative stress [35]. The ubiquitin kinase, PINK1, is recruited to the outer mitochondrial membrane to mediate ubiquitin phosphorylation and initiate mitophagy. PINK1 recruits Parkin, the E3 ubiquitin ligase, to build ubiquitin chains and assemble autophagy receptors, leading to mitophagy and reduced mtROS production [36-38]. In IBD, mitophagy is disrupted, mtDNA is damaged, and the mtDNA copy number is reduced, causing ROS accumulation, which leads to oxidative stress [29, 39, 40]. Abnormal ROS levels lead to 8-OHDG production [41]. In turn, the accumulated 8-OHDG activates NLRP3 and induces IL-1β secretion in mice [42]. Our laboratory previously found that, under HTH conditions, mice exhibited lower intestinal mtDNA levels, higher P62 levels, and lower LC3II levels compared to control mice, suggesting that the inhibition of autophagy may be responsible for the reduced mtDNA copy number [43]. Results of this study showed that the Parkin expression level and mtDNA copy number were lower, and 8-OHDG was overproduced in the mice in the HTH group compared with those in the NC group. HTH conditions increase NLRP3 inflammasome activation through downregulating mitophagy in mice, leading to mitochondrial damage and oxidative stress. In addition, the mice did not recover from the symptoms 1 week after their removal from the HTH environment, as observed on day 35, which may have been related to the lingering effects of the HTH environment, as described in traditional Chinese medicine.

Palmatine is an isoquinoline alkaloid that exhibits anti-inflammatory and antioxidant activities. A combination of network pharmacology and an experimental approach was used to determine the therapeutic mechanism through which palmatine acts in treating IBD. One hundred eighty-three targets of palmatine were identified in IBD using network pharmacology. The results of the analyses of the GO BPs, KEGG enrichment, and Cytoscape network relationship revealed that palmatine regulated the inflammatory response, oxidative damage, autophagy, and other aspects. The protective effect of palmatine against HTH-induced enteritis in animal experiments was evaluated. The results showed that palmatine treatment after HTH-induced enteritis led to gradual weight recovery, reduced the faecal water content, reduced inflammatory cell infiltration, improved the pathological status of the mice with enteritis, and inhibited the secretion of inflammatory cytokines, such as NLRP3 and IL-1β. These results show that palmatine may be suitable for treating HTH-induced enteritis in mice. However, palmatine administration triggered mitophagy *via* PI3K-AKT signalling through regulating Parkin. Palmatine ameliorated mitochondrial dysfunction through increasing the mtDNA copy number, reducing 8-OHDG levels, reducing oxidative stress markers in the intestinal tissues, and alleviating intestinal tissue damage. The findings demonstrate the beneficial effects of palmatine on mitophagy and intestinal inflammation in mice under HTH conditions.

An inhibitor of mitophagy, CsA, was used to further investigate whether palmatine inhibits NLRP3 inflammasome activation *via* modulating mitophagy. CsA suppresses the immune responses that modulate mitophagy [44]. The results showed that CsA administration blocked the expression of Parkin as well as decreased the mtDNA copy number and mtDNA damage. Additionally, CsA administration reversed the effect of palmatine on inhibiting NLRP3 inflammasome activation. The network pharmacology predictions were further confirmed in this study, which indicated that palmatine primarily alleviates enteritis by promoting autophagy, reducing mitochondrial damage, inhibiting oxidative stress, and decreasing the protein levels of NLRP3 and IL-1β. Palmatine may be a therapeutic target for inhibiting the NLRP3 activation associated with inflammatory stress, providing a novel strategy for alleviating HTH-induced enteritis in mice through regulating the autophagy-NLRP3 inflammasome axis. In addition, various indices minimally differed between the end of treatment and 1 week after drug withdrawal, but the differences in the NLRP3 and IL-1β levels increased between these time points, indicating that palmatine continued to exert effects 1 week after drug withdrawal but with delayed efficacy. In addition, the LD50 of palmatine in acute toxicity tests in mice is 1.534 g/kg, which is 15 times higher than the highest dose used in this study. Administering palmatine (521 mg/kg) for 90 days did not result in death or any signs of toxicity in rats [14]. Thus, the palmatine dose used in this study can be considered safe.

## CONCLUSION

HTH conditions induced enteritis and exacerbated enteritis-associated damage, potentially through inactivating the NLRP3 inflammasome mediated by the inhibition of Parkin-driven mitophagy. Palmatine treatment ameliorated the HTH-induced ileal damage and reduced intestinal inflammation. Palmatine may attenuate the symptoms of mild enteritis induced by an HTH environment through promoting Parkin-mediated mitophagy, reducing mitochondrial damage, reducing oxidative stress, and mediating NLRP3 inflammasome inactivation. Palmatine may be suitable as a multitarget, multi-pathway treatment for IBD. The results show that mitophagy and the NLRP3 inflammasome can be targeted for treating IBD through demonstrating the protective effects of palmatine against HTH-induced enteritis. These findings are consistent with those of previous research, indicating the role of mitophagy in regulating inflammation and highlighting palmatine as a promising therapeutic agent for treating IBD. The pharmacological mechanisms of action of palmatine against IBD identified in this study require further research, as several aspects need to be clarified. For example, the public databases may not have included some compounds or target genes due to the general nature of network pharmacology studies and their limitations. Further animal studies are needed to determine the specific mechanisms underlying our observations, including how HTH conditions inhibit mitophagy and how palmatine regulates mitophagy. Male mice were used to avoid the potential confounding effects of the oestrous cycle, which limits the generalizability of the findings to female mice. The BALB/C mouse model, although useful for studying inflammatory responses, may not fully replicate the complexity of human IBD. The variability in the experimental conditions, such as temperature and humidity, could have led to errors in the results. Consistent conditions were maintained, still minor variations were inevitable. This study indicates the therapeutic potential of palmatine. However, further research is needed to address the limitations of this study and validate the findings across different models and conditions. The long-term effects of palmatine, its pharmacokinetics, and potential side effects in humans need to be investigated for developing palmatine as a therapeutic agent. The potential genes targeted by palmatine in IBD were screened through database predictions and identified several overlapping genes, suggesting that palmatine exerts effects through regulating oxidative stress, inflammation, and autophagy pathways. Future functional studies, using siRNA or CRISPR to target Parkin or NLRP3, are needed to further validate the direct targets involved in these regulatory processes.

## AUTHORS’ CONTRIBUTIONS

The authors confirm their contribution to the paper as follows; analysis and interpretation of results — XYM, WLL, TM, YW, validation — XYM, TM, YLW, XHH, draft manuscript — YXM, SC, HHL. All authors reviewed the results and approved the final version of the manuscript.

## Figures and Tables

**Fig. (1):**
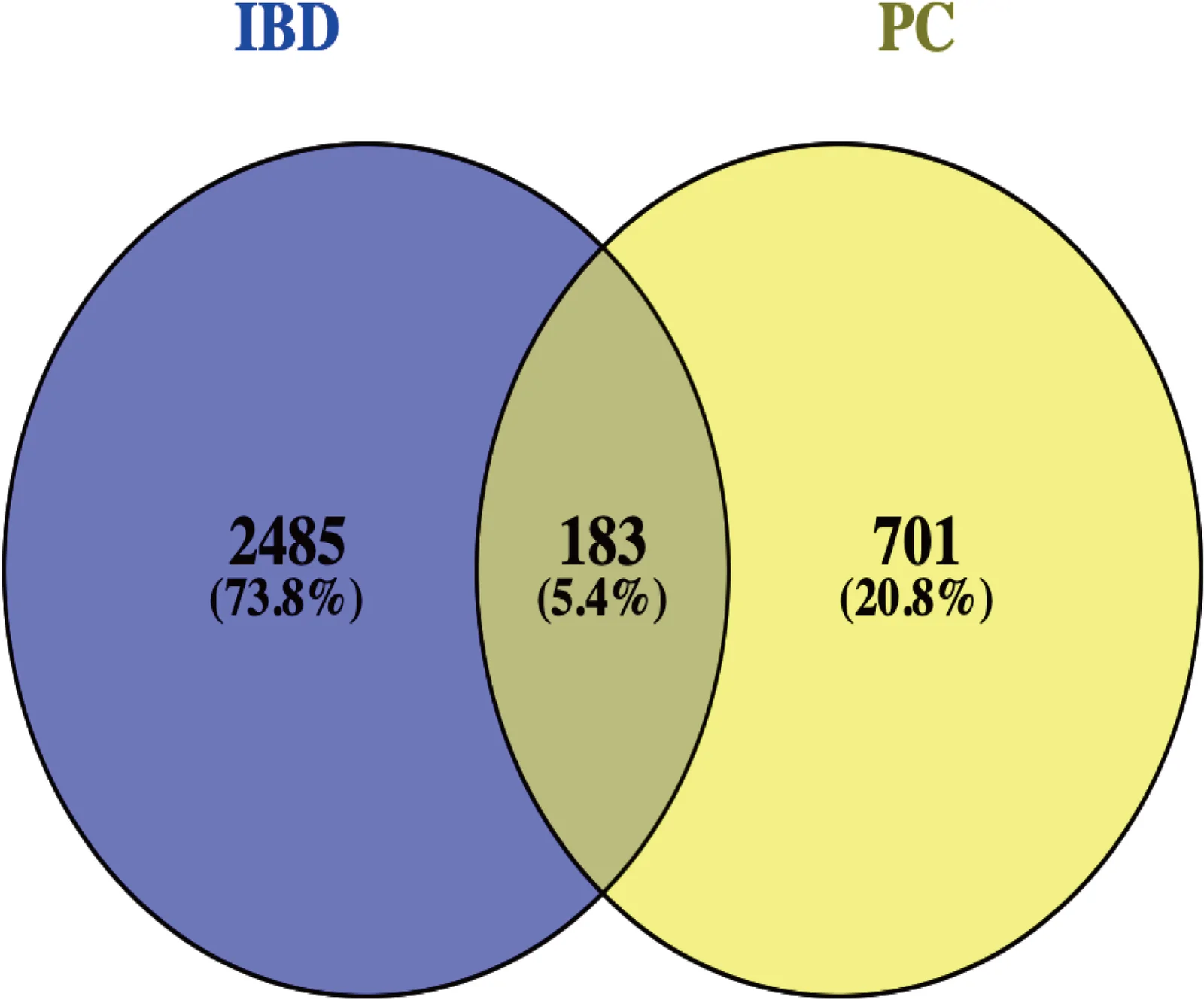
Venn diagram of the common targets of palmatine and inflammatory bowel disease (IBD).

**Fig. (3):**
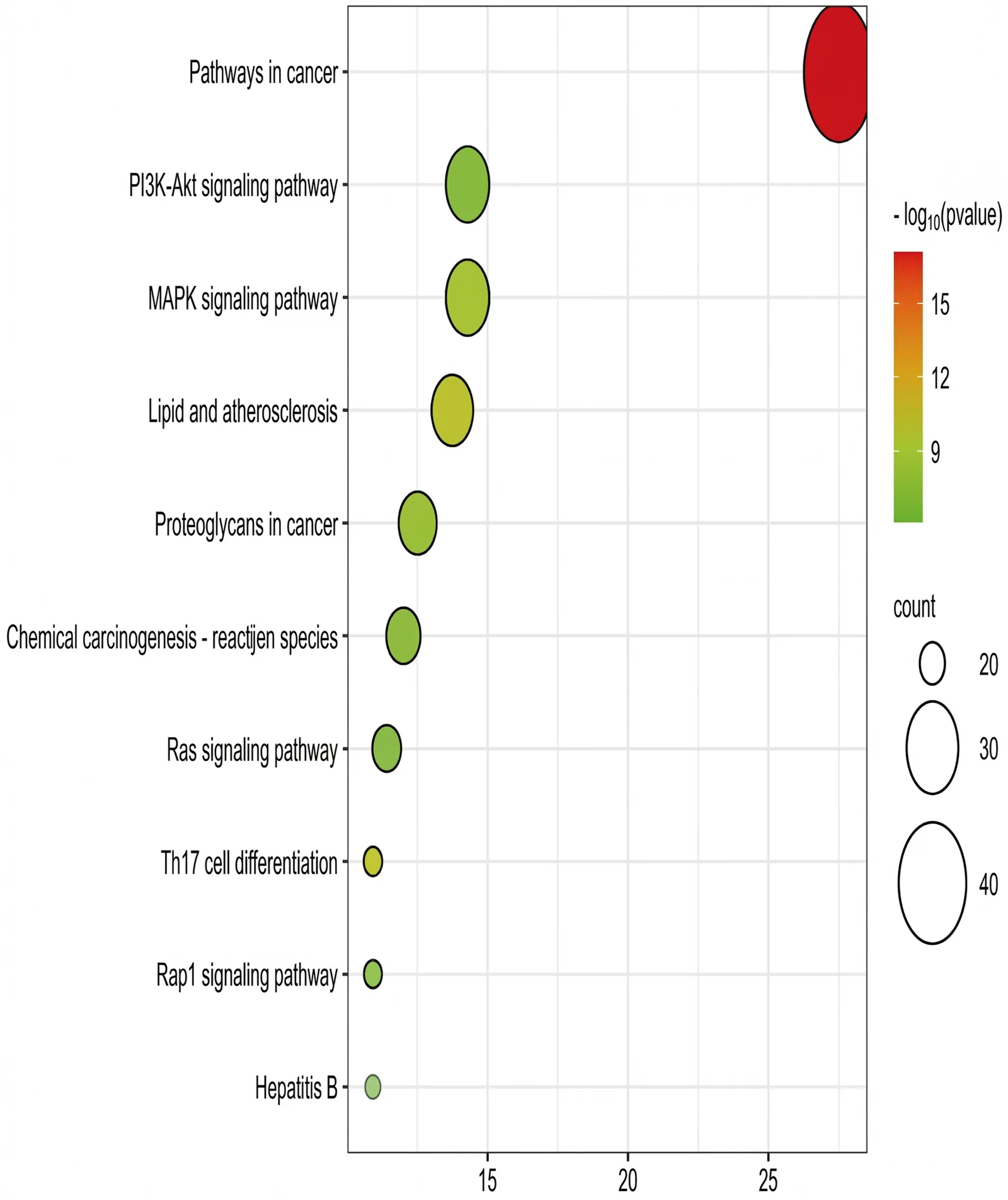
Top 10 signalling pathways for treating IBD with palmatine.

**Fig. (6):**
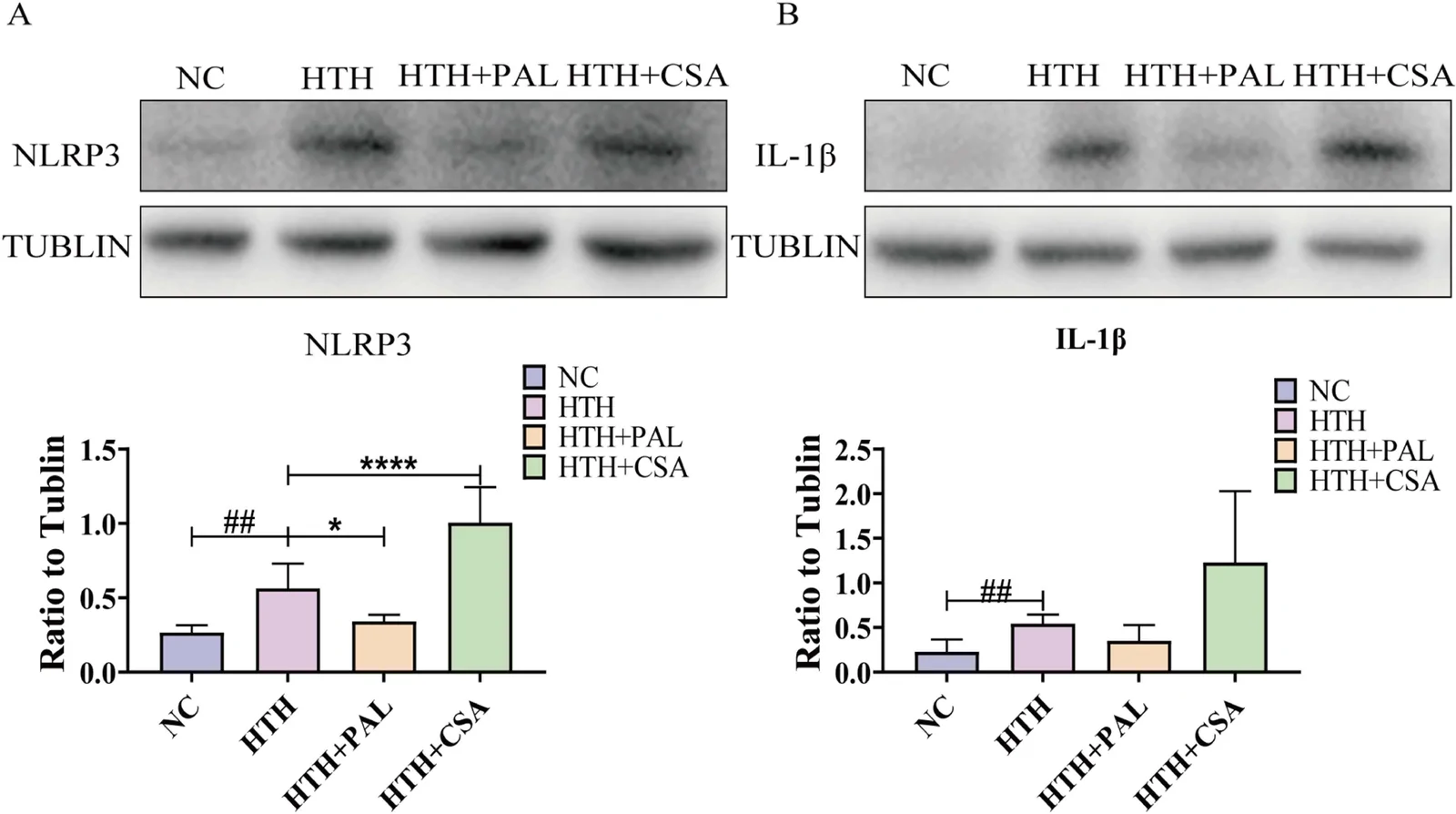
Palmatine attenuated the inflammatory response and inhibited the activation of NLRP3 and IL-1β in mice under HTH conditions. (**A**) NLRP3 and (**B**) IL-1β protein levels in ileal tissues (n=6). #: *P* < 0.01 *vs*. the NC group; *: *P* < 0.05 *vs*. the HTH group; ****: *P* < 0.0001 *vs*. the HTH group. Mean± standard deviation, one-way ANOVA, Kruskal-Wallis test.

## Data Availability

The datasets used and/or analysed in the current study will be available from the first author upon reasonable request.

## LIST OF ABBREVIATIONS

8-OHdG: 8-hydroxy 2’-deoxyguanosine
ASC: Apoptosis-associated speck-like protein containing a caspase recruitment domain
BPs: Biological Processes
CsA: Cyclosporin A
DAPI: 4′,6-diamidino-2-phenylindole
DAVID: Database for Annotation, Visualisation, and Integrated Discovery
GO: Gene Ontology
HTH: High Temperature And Humidity
IBD: Inflammatory Bowel Disease
IL-1β: Interleukin-1β
KEGG: Kyoto Encyclopedia of Genes and Genomes
mtDNA: mitochondrial DNA
NC: Normal Control
NLRP3: Nod-like receptor family pyrin-domain-containing protein 3
OMIM: Online Mendelian Inheritance in Man
PBST: Phosphate-Buffered Saline with Tween 20
TNF-α: Tumour Necrosis Factor
