# Supplementary material for: Palmatine Ameliorates High-Temperature and High-Humidity-Induced Enteritis via Mitophagy and NLRP3 Inflammasome Degradation
Source: Endocr Metab Immune Disord Drug Targets. 2025 Jul 15;26:E18715303348448. doi: 10.2174/0118715303348448250605071743 (PMC13523179; doi:10.2174/0118715303348448250605071743)
Supplement: Supplementary file 1 [file EMIDDT-26-E18715303348448_SD1.pdf]

***Endocrine, Metabolic & Immune Disorders - Drug Targets*, 2026, *Vol. 26*, e18715303348448 i**

Supplementary Material

Palmatine Ameliorates High-Temperature and High-Humidity-Induced Enteritis *Via* Mitophagy and NLRP3 Inflammasome Degradation

Xiaoying Mo[1,#], Tai Mi[2,#], Wanli Liu[3,#], YaoWang[3], Yalan Wu[3], Xinhua Huang[3], Song Chen[4,*] and Huanhuan Luo[3,5,*]

[1]College of Traditional Chinese Medicine, Changsha Medical University, Changsha 410219, Hunan Province, China; [2]Changsha Medical University, College of Traditional Chinese Medicine, Changsha, China; Present Address: Taoyuan Hospital of Traditional Chinese Medicine, Changde 415700, Hunan Province, China; [3]School of Basic Medicine, Guangzhou University of Chinese Medicine, Guangzhou 510006, Guangdong Province, China; [4]Science and Technology Innovation Center of Guangzhou University of Chinese Medicine, Guangzhou 510006, Guangdong Province, China; [5]State Key Laboratory of Dampness Syndrome of Chinese Medicine, Guangzhou, Guangdong 510006, China

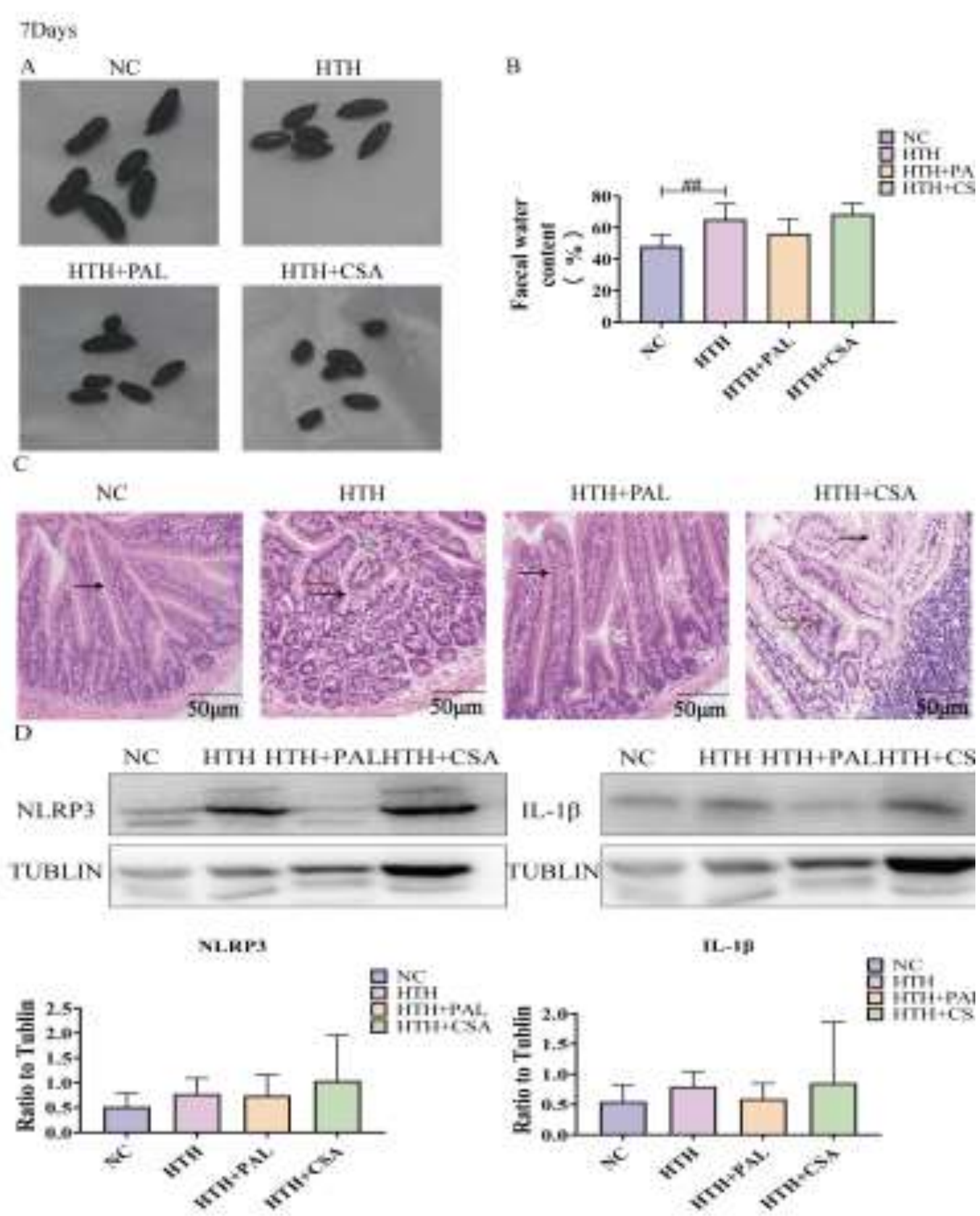

Additional Figure 1. Mice under High Temperature and Humidity (HTH) conditions were treated with palmatine continuously for 7 days. The appearance of the faeces, faecal water content, histological changes in the ileum, and NLRP3 and IL-1β protein levels were then evaluated. [a] Appearance of faeces. [b] Changes in the faecal water content (n = 6). [c] Representative haematoxylin and eosin staining images of ileal tissue (scale bar, 50 μm). [d] NLRP3 protein levels ileal tissues. [e] IL-1β protein levels in ileal tissues.

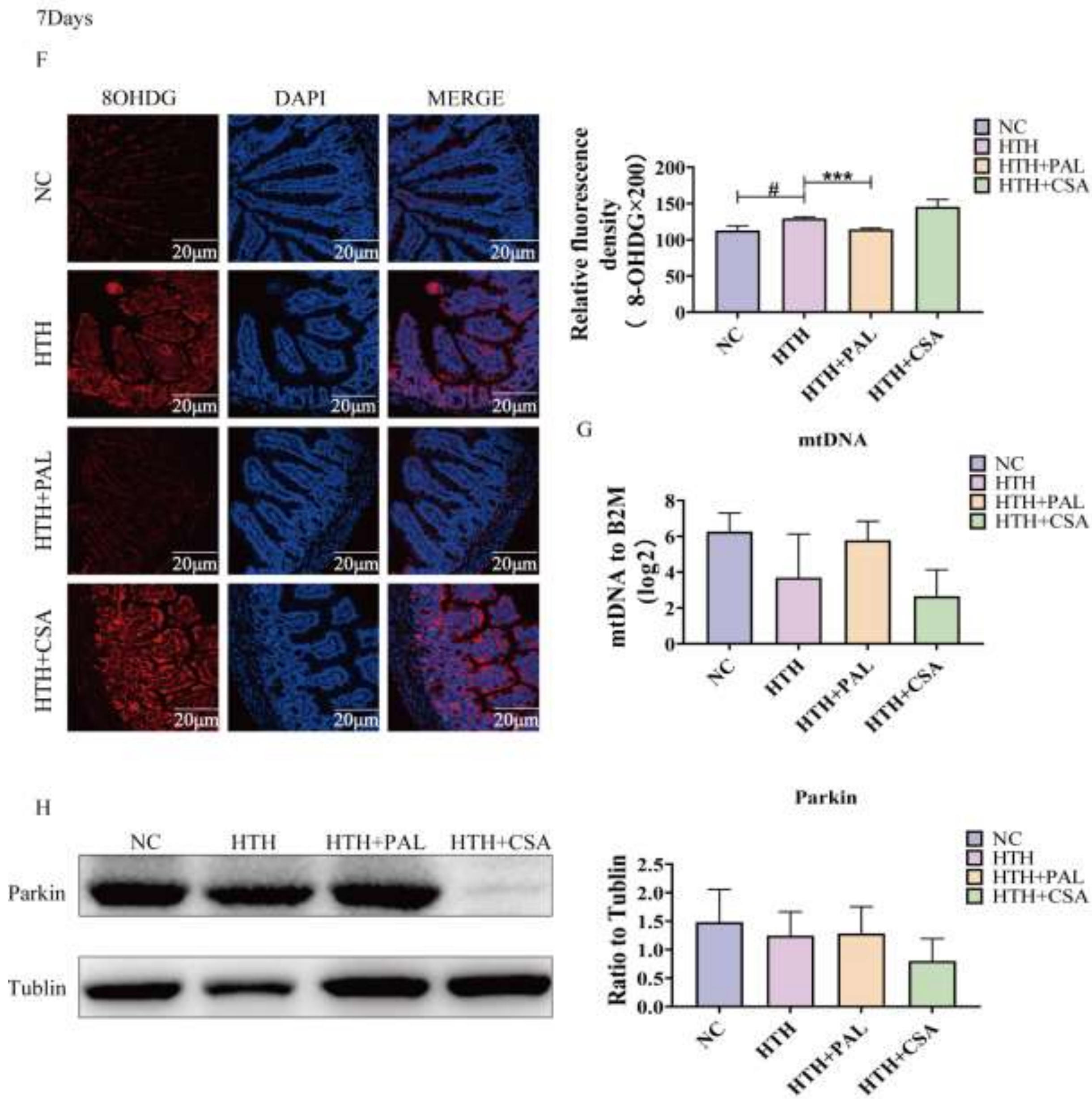

Additional Figure 2. Mice under HTH conditions were treated with palmatine continuously for 7 days. Palmatine promoted Parkin-mediated mitophagy and reduced mitochondrial DNA (mtDNA) damage and 8-OHDG levels in mice under HTH conditions. [f] Ileal tissues were stained with DAPI (blue), immunostained for 8-OHDG (red), and observed under a confocal laser-scanning microscope (scale bar, 20 μm; n = 4). [g] Polymerase chain reaction detection of mtDNA copy number in ileal tissues (n = 6). [h] Analysis of Parkin protein levels in mouse ileal tissues by western blotting (n = 6). #: P < 0.05 vs. the normal control group; ***: *P* < 0.001 vs. the HTH group.

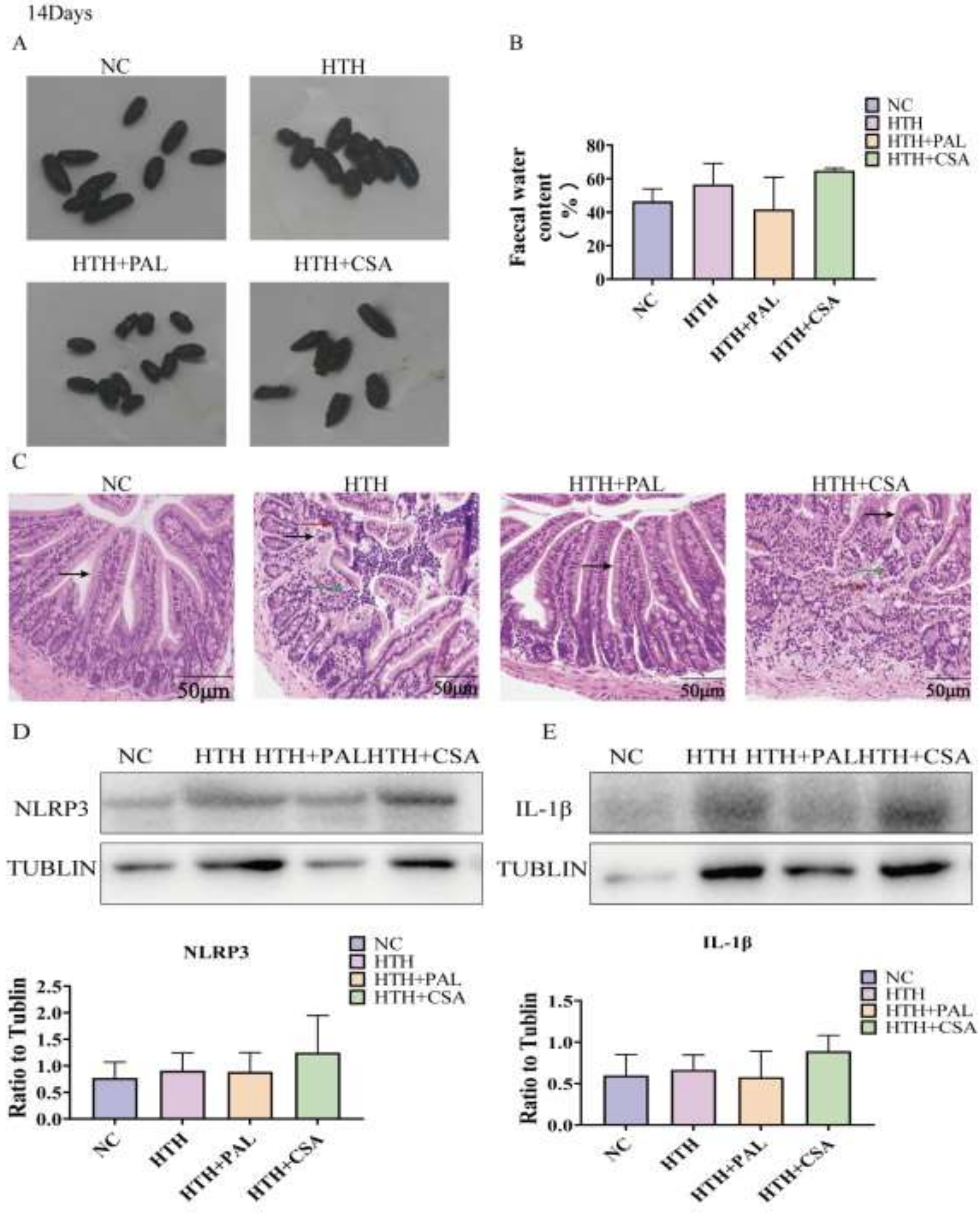

Additional Figure 3. Mice under High Temperature and Humidity (HTH) conditions were treated with palmatine continuously for 14 days. The appearance of the faeces, faecal water content, histological changes in the ileum, and NLRP3 and IL-1β protein levels were then evaluated. [a] Appearance of faeces. [b] Changes in the faecal water content (n = 6). [c] Representative haematoxylin and eosin staining images of the ileal tissue (scale bar, 50 μm). [d] NLRP3 protein levels in ileal tissues. [e] IL-1β protein levels in ileal tissues.

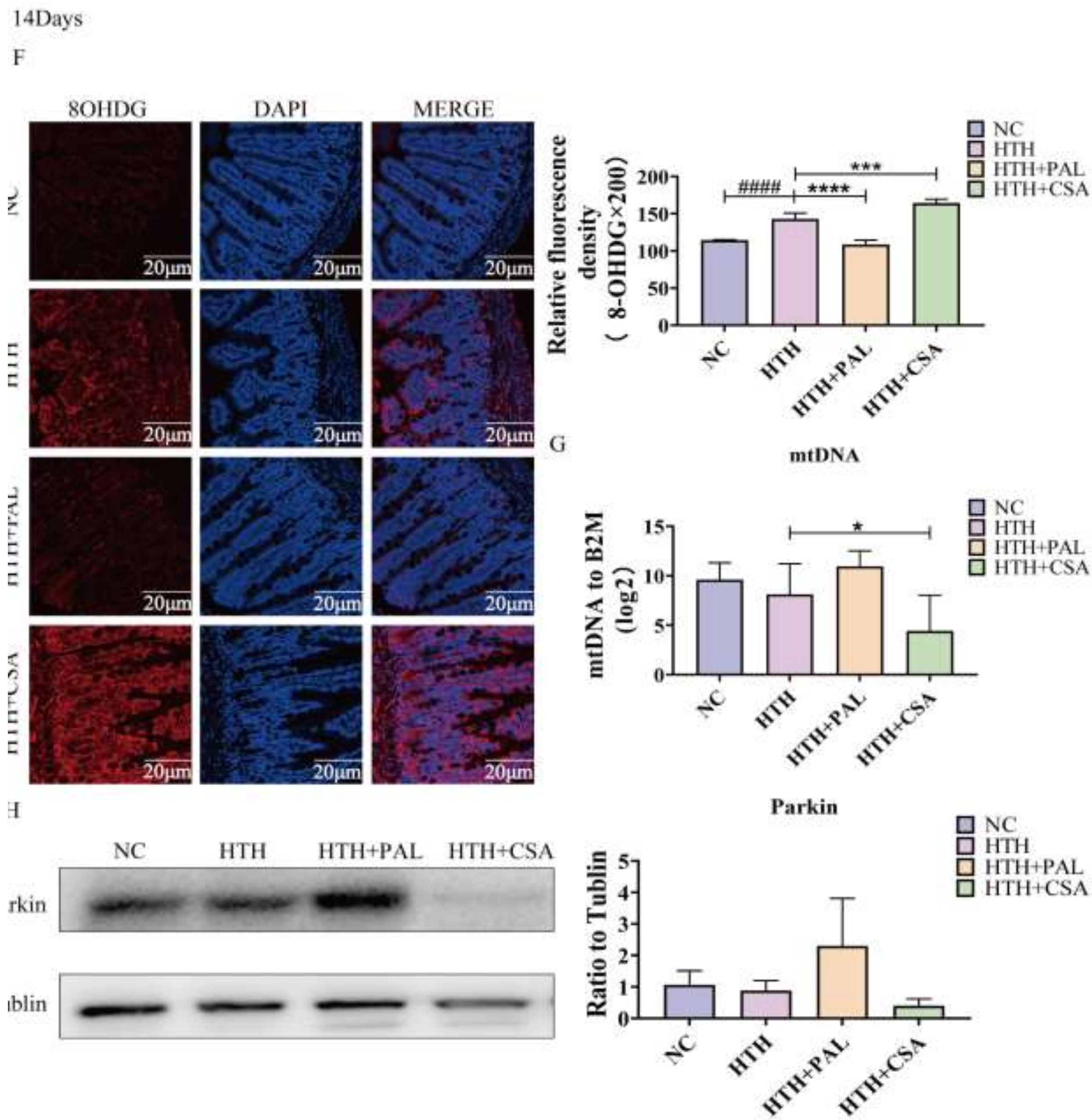

Additional Figure 4. Mice under HTH conditions were treated with palmatine continuously for 14 days. Palmatine promoted Parkin-mediated mitophagy and reduced mitochondrial DNA (mtDNA) damage and 8-OHDG levels in mice under HTH conditions. [f] Ileal tissues were stained with DAPI (blue), immunostained for 8-OHDG (red), and observed under a confocal laser-scanning microscope (scale bar, 20 μm; n = 4). [g] Polymerase chain reaction detection of mtDNA copy number in ileal tissues (n = 6). [h] Analysis of Parkin protein levels in mouse ileal tissues by western blotting (n = 6). ####: P < 0.0001 vs. the normal control group; *, ***, ****:P < 0.05, P < 0.001, P < 0.0001 vs. the HTH group.

The ARRIVE Essential 10			
These items are the basic minimum to include in a manuscript. Without this information, readers and reviewers cannot assess the reliability of the findings.			
**Item**		**Recommendation**	**Section/line number, or reason for not reporting**
**Study design**	1	For each experiment, provide brief details of study design including:<br>a. The groups being compared, including control groups. If no control group has been used, the rationale should be stated.<br>b. The experimental unit (e.g. a single animal, litter, or cage of animals).	Methods Section<br>Methods Section
**Sample size**	2	a. Specify the exact number of experimental units allocated to each group, and the total number in each experiment. Also indicate the total number of animals used.<br>b. Explain how the sample size was decided. Provide details of any *a priori* sample size calculation, if done.	2.2. Animal Model<br>2.2. Animal Model
**Inclusion and exclusion criteria**	3	a. Describe any criteria used for including and excluding animals (or experimental units) during the experiment, and data points during the analysis. Specify if these criteria were established *a priori*. If no criteria were set, state this explicitly.<br>b. For each experimental group, report any animals, experimental units or data points not included in the analysis and explain why. If there were no exclusions, state so.<br>c. For each analysis, report the exact value of *n* in each experimental group.	2.3. Ethical Approval<br>2.3. Ethical Approval<br>2.3. Ethical Approval
**Randomisation**	4	a. State whether randomisation was used to allocate experimental units to control and treatment groups. If done, provide the method used to generate the randomisation sequence.<br>b. Describe the strategy used to minimise potential confounders such as the order of treatments and measurements, or animal/cage location. If confounders were not controlled, state this explicitly.	2.3. Ethical Approval section.<br>2.2. Animal Model Development
**Blinding**	5	Describe who was aware of the group allocation at the different stages of the experiment (during the allocation, the conduct of the experiment, the outcome assessment, and the data analysis).	469-472<br>linenumb
**Outcome measures**	6	a. Clearly define all outcome measures assessed (e.g. cell death, molecular markers, or behavioural changes).<br>b. For hypothesis-testing studies, specify the primary outcome measure, i.e. the outcome measure that was used to determine the sample size.	2.5. Faecal Water Content<br>5. Conclusions section.
**Statistical methods**	7	a. Provide details of the statistical methods used for each analysis, including software used.<br>b. Describe any methods used to assess whether the data met the assumptions of the statistical approach, and what was done if the assumptions were not met.	2.10. Statistical Analysis<br>2.10. Statistical Analysis

**Experimental animals**	8	a. Provide species-appropriate details of the animals used, including species, strain and substrain, sex, age or developmental stage, and, if relevant, weight.<br>b. Provide further relevant information on the provenance of animals, health/immune status, genetic modification status, genotype, and any previous procedures.	2.2. Animal Model<br>2.2. Animal Model
**Experimental procedures**	9	For each experimental group, including controls, describe the procedures in enough detail to allow others to replicate them, including:<br>a. What was done, how it was done and what was used.<br>b. When and how often.<br>c. Where (including detail of any acclimatisation periods).<br>d. Why (provide rationale for procedures).	2.3. Ethical Approval<br>2.3. Ethical Approval ETHICS APPROVAL<br>HTH Induction: The
**Results**	10	For each experiment conducted, including independent replications, report:<br>a. Summary/descriptive statistics for each experimental group, with a measure of variability where applicable (e.g. mean and SD, or median and range).<br>b. If applicable, the effect size with a confidence interval.	Detailed data is listed in a<br>Detailed data is listed in a

NC 3Rs National Centre for the Replacement Refinement & Reduction of Animals in Research

The Recommended Set			
These items complement the Essential 10 and add important context to the study. Reporting the items in both sets represents best practice.			
**Item**		**Recommendation**	**Section/line number, or reason for not reporting**
**Abstract**	11	Provide an accurate summary of the research objectives, animal species, strain and sex, key methods, principal findings, and study conclusions.	Abstract section
**Background**	12	a. Include sufficient scientific background to understand the rationale and context for the study, and explain the experimental approach.<br>b. Explain how the animal species and model used address the scientific objectives and, where appropriate, the relevance to human biology.	1. INTRODUCTI<br>BALB/c mice were chosen
**Objectives**	13	Clearly describe the research question, research objectives and, where appropriate, specific hypotheses being tested.	
**Ethical statement**	14	Provide the name of the ethical review committee or equivalent that has approved the use of animals in this study, and any relevant licence or protocol numbers (if applicable). If ethical approval was not sought or granted, provide a justification.	ETHICS APPROVAL AND CONSENT TO
**Housing and husbandry**	15	Provide details of housing and husbandry conditions, including any environmental enrichment.	2.2. Animal Model Development section. 2.3.

**Animal care and monitoring**	16	a. Describe any interventions or steps taken in the experimental protocols to reduce pain, suffering and distress.<br>b. Report any expected or unexpected adverse events.<br>c. Describe the humane endpoints established for the study, the signs that were monitored and the frequency of monitoring. If the study did not have humane endpoints, state this.	2.3. Ethical Approval<br>Expected Adverse<br>Humane Endpoints: The
**Interpretation/ scientific implications**	17	a. Interpret the results, taking into account the study objectives and hypotheses, current theory and other relevant studies in the literature.<br>b. Comment on the study limitations including potential sources of bias, limitations of the animal model, and imprecision associated with the results.	CONCLUSION<br>Despite the promising
**Generalisability/ translation**	18	Comment on whether, and how, the findings of this study are likely to generalise to other species or experimental conditions, including any relevance to human biology (where appropriate).	CONCLUSION
**Protocol registration**	19	Provide a statement indicating whether a protocol (including the research question, key design features, and analysis plan) was prepared before the study, and if and where this protocol was registered.	A detailed research protocol,
**Data access**	20	Provide a statement describing if and where study data are available.	Provide a statement describing if and where s
**Declaration of interests**	21	a. Declare any potential conflicts of interest, including financial and non-financial. If none exist, this should be stated.<br>b. List all funding sources (including grant identifier) and the role of the funder(s) in the design, analysis and reporting of the study.	CONFLICT OF INTEREST<br>FUNDING<br>section.

www.ARRIVEguidelines.org
